# Chinosol

**Published:** 1899-05

**Authors:** 


CHINOSOL. The antiseptic, disinfectant, deodorizer, and bactericide is still before the medical profession, but particularly before the veterinary profession. Dr. F. Hobday, of the Royal Veterinary College of London, England, discusses the therapeutical and toxicological effects of this agent. He has made extensive use of it during the previous nine months, both in his canine and equine clinics. It was used as an antiseptic on wounds of all kinds, in the form of solutions varying from 1 in 60 to 1 in 1200, with the most satisfactory results. The strength found to give the best results was from 32 to 65 milligrammes ($\frac{1}{2}$ to 1 grain) to 30 c.c. (1 fluidounce). Upon fetid and ulcerating wounds a proportion of 1 in 480 speedily caused a healthy appearance and entire absence of pus after application once or twice per day. In treating a wound in different parts of the same animal opportunities were often gained to note the effects of the solution of this strength in comparison with those of lysol and creolin, with the result that chinosol gave decidedly the best results. As a dry dressing in the form of powder, when mixed with either boric acid, zinc oxide, or starch, and then compared with iodoform used in a similar manner, the sequela appeared to be about the same. As a disinfectant to the hands, skin, and suture threads, a solution was used varying from 1 to 1000 to 1 to 60, without any signs of irritation either to the hands of the operator or the skin of the patient. Care must be taken, however, with instruments, as on several occasions Dr. Hobday reports that the instruments lost their edge and the steel parts became coated with greenish-black spots, which were troublesome to remove, and in those instruments that had white bone handles the latter became discolored and rough to the touch. The solution, therefore, recommended for such purposes consists of 1 in 1200, and if the instruments are to remain in it for anything like an hour, this strength should certainly not be exceeded. As a deodorizer for the hands or for fetid wounds, solutions of the same strength as those used for disinfectant purposes act satisfactorily. Details of several illustrative cases were related, and the author sums up the conclusions to which his experience has led him as follows:

"(1) That chinosol acts well as an antiseptic, disinfectant, and deodorant when used in certain proportions. (2) That its action is better marked when used as a lotion than when used as a powder. (3) That the powder is not suitable on fresh wounds unless diluted in some way or other. (4) That for the disinfection of instruments care must be taken not to make the solution too concentrated. (5) That the drug possesses toxic properties. (6) That if used subcutaneously in too concentrated a form it will produce local irritation and swelling. The strength recommended for subcutaneous injection in human practice is from 1 in 600 to 1 in 200. (7) That the cat is very susceptible to its action, and that in this animal much more care is necessary to guard against toxic symptoms than in the case of the dog. In the cat, if subcutaneously injected, the extreme limit of dose should be one-sixteenth of a grain for each pound of body-weight; and in the dog, one-eighth of a grain per pound. (8) That chinosol is not rapidly absorbed from the unbroken skin of the dog, and can be applied for several days in succession, even in fairly concentrated solution, to the skin of this animal without producing eruptions or sores. (9) That the chief symptoms of poisoning are: Sneezing and coughing, an increased flow of thick, ropy saliva, subnormal temperature, staggering gait, commencing with loss of motor power in the hind quarters; great prostration, and ultimate death from failure of the heart's action. (10) That the chief post-mortem characteristic is the smell of chinosol on or in some part of the body, while another symptom to be looked for is the presence of frothy saliva in the pharynx, œsophagus, or stomach.—*Epitome of British Medical Journal*, 1898, vol. i. p. 91.

